# Tackling regulated cell death yields enhanced protection in lung grafts

**DOI:** 10.7150/thno.87375

**Published:** 2023-07-31

**Authors:** Qian Chen, Xiangfeng Liu, Zhiheng Liu, Shujing Zhang, Lin Chen, Shiori Eguchi, Azeem Alam, Zhen Cahilog, Qizhe Sun, Lingzhi Wu, Enqiang Chang, Zhiping Wang, Jianteng Gu, Hailin Zhao, Daqing Ma

**Affiliations:** 1 Division of Anaesthetics, Pain Medicine and Intensive Care, Department of Surgery and Cancer, Faculty of Medicine, Imperial College London, Chelsea & Westminster Hospital, London, UK.; 2 Department of Anaesthesiology, Southwest Hospital, Army Medical University, Chongqing, China.; 3 Children's Hospital, Zhejiang University School of Medicine, National Clinical Research Center for Child Health, Zhejiang, China.; 4 Department of Anaesthesiology, Shenzhen Second People's Hospital/the First Affiliated Hospital of Shenzhen University, Health Science Centre, Shenzhen, China.; 5 Department of Anaesthesiology, Affiliated Hospital of Xuzhou Medical University, Xuzhou, Jiangsu, China.

**Keywords:** Donation after cardiocirculatory death, Lung transplantation, Ischaemia reperfusion injury, Regulated cell death, Dexmedetomidine.

## Abstract

**Background:** Effective preservation strategies to ameliorate lung graft ischaemia injury are needed to rescue 'extended criteria' or 'marginal' lung grafts, and to improve recipient outcomes after transplantation.

**Methods:** Lung grafts from male Lewis rats were extracted after 40 min of cardiocirculatory death, and healthy human lung tissues were collected from patients undergoing a lobectomy. Lung samples were then preserved in a 4°C preservation solution supplemented with 0.1 nM Dexmedetomidine (Dex, α_2_-adrenoceptor agonist) for 16 h. *In vitro*, human lung epithelial A549 cells were preserved in the 4°C preservation solution with 0.1 nM Dex for 24 h, then re-cultured in the cell culture medium at 37°C to mimic the clinical scenario of cold ischaemia and warm reperfusion. Lung tissues and cells were then analysed with various techniques including western blot, immunostaining and electron microscope, to determine injuries and the protection of Dex.

**Results:** Prolonged warm ischaemia after cardiocirculatory death initiated Rip kinase-mediated necroptosis, which was exacerbated by cold storage insult and enhanced lung graft injury. Dex supplementation significantly reduced necroptosis through upregulating Nrf2 activation and reducing oxidative stress, thereby significantly improving lung graft morphology. Dex treatment also attenuated endoplasmic reticulum stress, stabilised lysosomes and promoted cell membrane resealing function, consequently reducing cell death and inflammatory activation after hypothermic hypoxia-reoxygenation in A549 cells.

**Conclusions:** Inhibition of regulated cell death through Dex supplementation to the graft preservation solution improves allograft quality which may aid to expand the donor lung pool and enhance lung transplant outcomes *per se*.

## Introduction

Despite improvements in surgical management, perioperative care and immunosuppression, the outcome of lung transplantation remains the worst amongst solid organ transplant recipients, with a median survival of only 5.8 years 1, 2. Meanwhile, the demand for lung transplantation continued to outpace donations, as reported a 42.2% increase on the waiting list over the past decade 3. Lung graft deficit comprises a small number of donors and an extremely low rate (15-20%) of lung grafts suitable for transplanted 4. It has been known that graft survival and the success of transplantation are significantly limited by ischaemia reperfusion (IR) injury, which is recognised as a major determinant of primary graft dysfunction (PGD) and contributes to early morbidity and mortality 5, 6. Therefore, exploring novel therapies to attenuate IR injury is urgently needed to tackle an already depleted lung donor pool with a simultaneously increasing demand for lung grafts.

The use of 'Extended Criteria' or 'marginal' donors, such as lungs from donation after cardiocirculatory death (DCD) donors, is one of the options to tackle organ shortages. However, warm ischaemia-induced deterioration of cardiorespiratory function before and after cardiac arrest is recognised to damage lung grafts. Hypoxia and hypotension associated with warm ischaemia lead to systemic hemodynamic disturbances and metabolic abnormalities resulting in lung dysfunction and inflammation 7-9 whilst prolonged *ex vivo* cold preservation results in cold ischaemia to grafts. In response to hypoxia during cold preservation, mitochondria, the prominent organelles generating adenosine triphosphate (ATP), produce oxygen free radicals and lactic acid due to glycolytic substrates depletion, further damaging lung grafts 10, 11. Overall, damage to lung grafts before transplantation reduces lung survival and results in deleterious sequelae that precipitate subsequent graft dysfunction. Thus, therapeutic strategies that mitigate lung graft ischaemia injury may yield significant clinical benefits.

IR injury induces several distinct forms of cell death, of which the most prevalent forms of regulated cell death in transplantation are necroptosis 12, 13 and ferroptosis 14, 15. The critical characteristic of regulated cell death, such as necroptosis, is plasma membrane accidental disruption. After the loss of plasma membrane integrity, the cells continue de novo synthesis of damage-associated molecular patterns (DAMPs) and immunoregulatory molecules for several hours that, in turn, trigger inflammation and immunological response in the body 16, 17, which may increase the risk of PGD and rejection after transplantation. Therefore, elucidating the molecular mechanisms underpinning regulated cell death may facilitate the development of treatment strategies for improving patient outcomes in lung transplantation.

Previously, *in vitro* and *in vivo* studies demonstrated that an α_2_-adrenoceptor agonist, dexmedetomidine (Dex), exerts protective effects against IR injury of the liver, lung, and kidney 18-21, as well as being able to alleviate regulated cell death induced by hypoxia-reoxygenation 22. Therefore, this study aimed to determine whether a graft preservation solution with supplementation of Dex protects lung grafts by reducing regulated cell death, in particular necroptosis, and to explore the underlying molecular mechanisms in the setting of *ex vivo* lung grafts from rats and human lung tissues, as well as *in vitro* lung epithelial cell cultures.

## Methods

### Rat DCD donor and *ex vivo* lung graft preservation model

Inbred adult male Lewis rats (LEW, RT1^1^), weighing 225 - 250 g, purchased from Harlan, UK, were housed in temperature- and humidity-controlled cages in a specific pathogen-free facility at the Chelsea-Westminster Campus, Imperial College London, UK. All animal procedures were carried out in accordance with the United Kingdom Animals (Scientific Procedures) Act of 1986. As described previously, the cardiac arrest model (DCD donor) in anaesthetised rats was used 23. Briefly, donor rats were anaesthetised with an intraperitoneal injection of pentobarbital sodium (Abbott Laboratories, Chicago, USA) at 3.5 mg/100 g. Anticoagulation was accomplished by injecting 250 U Heparin in 1 mL saline *via* the penile vein. Five min after heparinisation, a thoracotomy was performed, followed by induction of cardiac arrest by external compression of the heart for 5 min. After the induction of cardiac arrest, the aorta was closed using a vascular clamp rostral from the heart. The thoracotomy wound was covered with gauze and kept moist with 0.9% NaCl. The entire lung was isolated and excised after 40 min (warm ischaemia period). A lung graft from a living donor (LD) was excised directly after terminal anaesthesia, which acted as the naïve control. All grafts were then flushed through the main pulmonary artery and stored at 4 °C for 16 h with either control University of Wisconsin (UW) solution (Bridge to Life Ltd, New York, USA) or UW solution containing Dex (0.1 nM) for further analyses.

### Human DCD lung graft model and lung cold preservation

After informed written consent was obtained, healthy lung tissues (> 5 cm far away from the lung tumour) were collected from 10 female patients (Table S1) with a diagnosis of lung tumour undergoing a lobectomy. The exclusion criteria were: a history of COPD or asthma, pulmonary hypertension, severe local or systemic infections, drug or alcohol abuse or long-term cigarette use, and any other medical co-morbidities that may confer increased risks. The excised lung tissue was inflated with warm saline, then cut into slices (1 x 2 x 0.5 cm), kept cold, and preserved in the UW solutions with or without 0.1 nM Dex for 16 h. No treatment lung tissues served as naïve controls.

### Administration of atipamezole and Nrf-2 siRNA to donor animals

Nrf-2 siRNA or scrambled siRNA (negative control) (Qiagen, Crawley, West Sussex, UK) were dissolved in siRNA suspension buffer and diluted in RNase-free PBS before use. Dissolved Nrf-2 or scrambled siRNA (200 µg in 10 mL of PBS) was rapidly injected (within 30 s) *via* a tail vein under anaesthesia, and rats were allowed to recover for 24 h before lung graft extraction 24. Atipamezole (α_2_-adrenergic antagonist, Sigma-Aldrich, St. Louis, United States, 250 μg/kg, i.p.) was administered 10 min before graft extraction 20.

### Administration of recombinant HMGB-1 protein

Donor rats received vehicle PBS or recombinant HMGB-1 protein (20 μg/kg, Abcam, Cambridge, UK) by intraperitoneal injection 24 h before lung extraction 25.

### Hematoxylin and eosin staining

Lung grafts were fixed in 4% parafamydehyde and paraffin. Sections of 5 µm thickness were taken from lung specimens, and hematoxylin & eosin (H&E) staining was carried out. Lung morphology in each graft (10 fields at x 20 magnifications) was evaluated by an observer blinded to the treatment using an Olympus BX4 microscope (Watford, UK) under a constant exposure level. The score for each field was calculated from the sum score of 10 areas chosen at random. Lung injury was categorised as Grade 0: normal appearance, negligible damage; Grade 1: mild-moderate interstitial congestion; Grade 2: perivascular oedema formation, partial destruction of pulmonary architecture; Grade 3: moderate lung alveolar damage, low level of nuclear fragmentation; Grade 4: severe destruction of the pulmonary architecture, high level of nuclear fragmentation or condensation 26.

### TUNEL staining

Lung alveolar cell death was detected with *in situ* TUNEL assay (Millipore, Burlington, United States) according to the instructions of the manufacturer. TUNEL^+^ nuclei were visualised by green FITC fluorescence.

### OxyIHC oxidative stress assay

5 µm lung sections were incubated in DNPH solution in the dark for 30 min and then blocked for 30 min at room temperature. The sections were then incubated with primary antibody (1:100) at 4 °C overnight and were then incubated with biotinylated secondary antibody for 1 h. After being incubated with the streptavidin-conjugated HRP for 30 min, the slides were incubated with the DAB-A/B mixture. The slides were examined using a Zeiss Axio Observer 7 microscope (Zeiss, Oberkochen, Germany) or an Olympus BX4 microscope (Watford, UK) under a constant exposure level.

### Enzyme-linked immunosorbent assay (ELISA)

Rat graft lung tissue TNF-α, IL-1b, and HMGB-1 were measured by ELISA (Rat TNF-α, IL-1b or HMGB-1 ELISA kits, Invitrogen, Waltham, United States) according to the instructions of the manufacturer.

### Determination of total glutathione (GSH and GSSG)

Total glutathione (GSH and GSSG) was measured in lung homogenates using a glutathione assay kit (Sigma-Aldrich, St. Louis, United States) according to the instructions of the manufacturer.

### *In vitro* cell culture and treatment

Human lung epithelial A549 cells (European Cell Culture Collection, UK) were cultured in RPMI 1640 medium supplemented with 10% fetal bovine serum (FBS), 2 mM L-glutamine, and 100 U/mL penicillin-streptomycin (all from Invitrogen, Waltham, United States). The cells were challenged with 150 ng/mL TNF-α (Sigma-Aldrich, St. Louis, United States), 10 μM SMAC mimetic and inhibitor of IAP (LCL161; MedChemExpress, UK), and 40 μM Q-VD-Oph (Sigma-Aldrich, St. Louis, United States) mixture as the standard necroptosis inducer 27. Other A549 cohort cultures were preserved in the UW solution with or without Dex (0.1 nM) and atipamezole (1 nM) at 4 °C for 24 h to simulate hypothermic preservation of lung grafts. These cultures were re-cultured in RPMI 1640 medium at 37 °C in the cell culture incubator to simulate warm reperfusion after engraftment.

### Western blot

Cell samples were mechanically homogenised in a lysis buffer. The lysates were centrifuged, and the supernatant was collected. The protein extracts (40 μg/sample) were heated, denatured, and loaded on a NuPAGE 4 to 12% Bis-Tris gel (Invitrogen, Waltham, United States) for electrophoresis and then transferred to a polyvinylidene difluoride membrane. The membrane was probed with primary antibodies (1:1000) purchased from (Cell Signaling Technology, Massachusetts, United States) or Abcam (Cambridge, UK) in TBS-T overnight at 4 °C, followed by HRP-conjugated secondary antibody (Cell Signaling Technology, Massachusetts, United States) for 1 h. The loading control was the constitutively expressed protein, Glyceraldehyde 3-phosphate dehydrogenase (GAPDH; 1:100000, Abcam, Cambridge, UK). The blots were visualised with the enhanced chemiluminescence system (SantaCruz, Dallas, United States) and analysed with GeneSnap (Syngene, Cambridge, UK).

### Immunohistochemistry

For *ex vivo* fluorescence staining, 5 µm lung sections were incubated in 4% normal donkey serum in 0.1 M PBS-T for 1 h and incubated with primary antibodies (1:200) purchased from Abcam (Cambridge, UK) or SantaCruz (Dallas, United States) at 4 °C overnight. After washing with PBS-T, the slides were incubated with fluorochrome-conjugated secondary antibodies (Millipore, Burlington, United States). For *in vitro* fluorescence staining, A549 cells were fixed in 4% paraformaldehyde and incubated in 4% normal donkey serum in 0.1 M PBS-T and subsequently incubated overnight with various primary antibodies (1:200 or 300) purchased from Abcam (Cambridge, UK) or SantaCruz (Dallas, United States), followed by a secondary antibody (1:500, Abcam, Cambridge, UK) for 1 h. For double-labelled immunofluorescent staining, lung sections and cell samples were incubated overnight with the first primary antibody, followed by the first secondary antibody, then the second primary antibody, and the second secondary antibody. The slides were counterstained with nuclear dye DAPI (Abcam, Cambridge, UK) and mounted with VECTASHIELD Mounting Medium (Vector Lab, USA). The slides were then examined using a Zeiss Axio Observer 7 microscope (Zeiss, Oberkochen, Germany) or an Olympus BX4 microscope (Watford, UK) under a constant exposure level. The immunofluorescent intensity was quantified using Image J (U.S. National Institutes of Health, Bethesda, MD). An assessor blinded to the treatment groups randomly selected 10 representative regions per section (*ex vivo*) or field (*in vitro*). Values were then calculated as percentages of the mean value of the naive controls and expressed as the percentage of controls.

### Intracellular calcium measurement

After preservation in cold UW solution for 24 h, A549 cells were incubated with 2 μM calcium indicator Fura-2 (Abcam, Cambridge, UK) in DMSO/HBSS for 30 min at room temperature in the dark, then re-cultured in fresh culture medium at 37 °C for up to 300 min. The cells were subsequently fixed with 4% paraformaldehyde and examined using an Olympus BX4 microscope (Watford, UK) under constant exposure time.

### Membrane lipid labelling

A549 cells were incubated with 5 μL/mL DilC18 (Molecular probes, New York, USA) for 20 min at 37 °C in the dark; after washing with PBS, the cells were preserved in UW solution with or without Dex (0.1 nM) and atipamezole (1 nM) at 4 °C for 24 h and then recultured in RPMI 1640 cell culture medium for another 6 h at 37 °C. The cells were fixed with 4% paraformaldehyde and examined using a Zeiss Axio Observer 7 microscope (Zeiss, Jena, Germany) under constant exposure time.

### Electron microscopy

Lung tissues from 3 patients were fixed in 2.5% glutaraldehyde to examine the morphology of the cytoplasm and nucleus of type II epithelial and endothelial cells. The samples and cellular sub-organelle injuries, such as the ratio of damaged mitochondria, the number of phagosome and mitophagosome-like structures, and the number of visible lamellar bodies in type II epithelial cells were counted and analysed by a specialist at the Electron Microscope Center of Third Military Medical University, Chongqing, China, who was blinded to the study design.

### Statistical analysis

All numerical data were expressed as mean ± standard deviation (SD) or median with interquartile range and presented with scatter plots. Data were analysed using the two-tailed Student's t-test or analysis of variance (ANOVA) followed by Kruskal-Wallis non-parametric (scoring) or Newman-Keuls (measurement) test for comparisons, where appropriate (GraphPad Prism 5.0, GraphPad Software, USA). A p value < 0.05 was considered to be statistically significant.

## Results

### Ischaemia induces lung graft injury and enhances inflammatory factors release in DCD lung grafts

**Figure 1:**
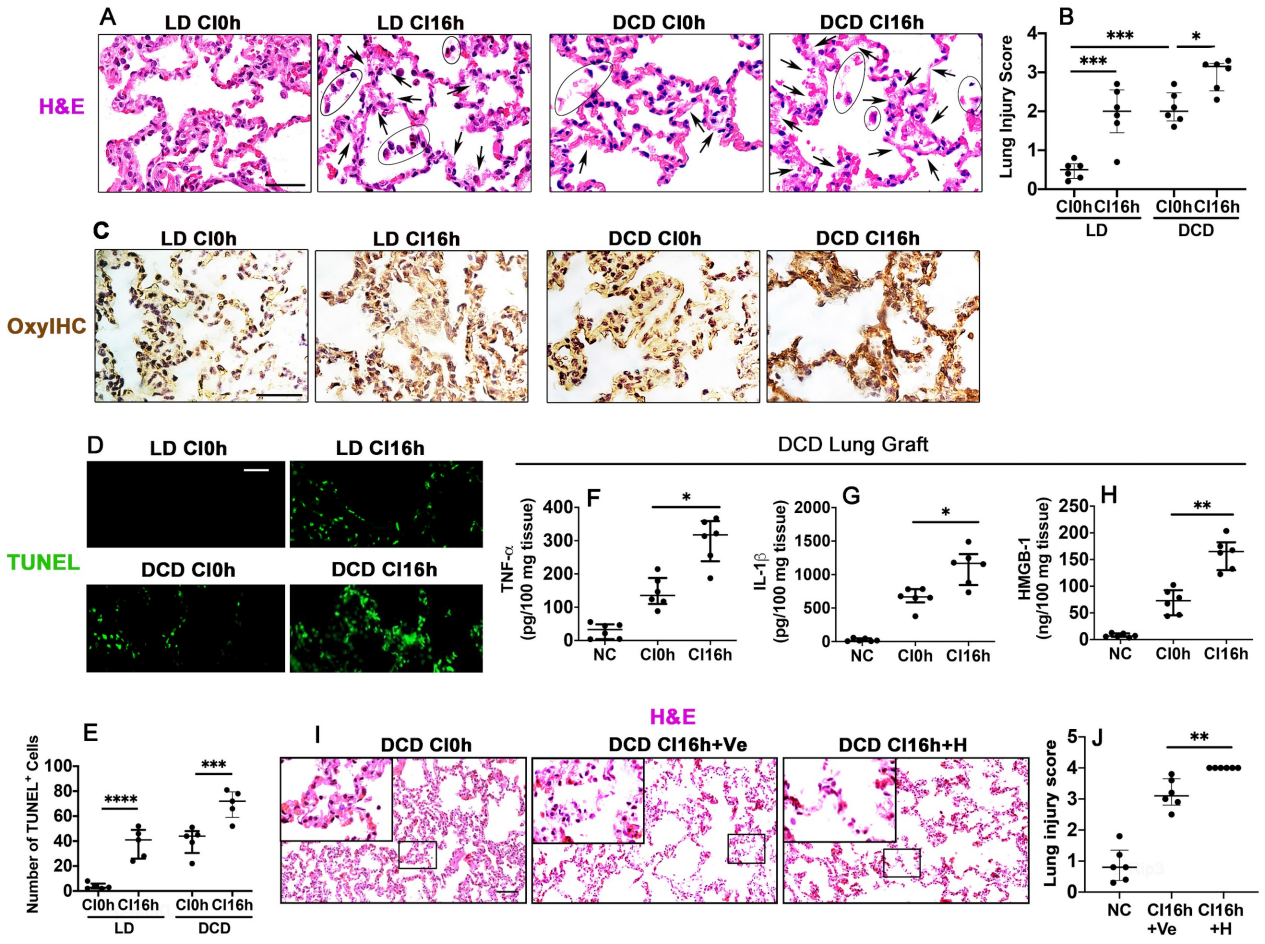
** Ischaemia induces lung graft injury and enhances inflammatory factors release in DCD lung grafts.** Illustration of experimental procedure of *ex vivo* lung grafts: Lung graft from a living donation (LD) rat was excised directly after terminal anaesthesia. The lung graft from a donation after cardiac death (DCD) rat was extracted after 40 min of cardiac arrest. Both lung grafts were flushed and stored in the 4 °C UW solution for 0 or 16 h (CI0h or CI16h). **(A)** Histology (H&E staining) of lung grafts. Arrows indicate coagulative necrosis and cell deterioration, and areas marked by black circles indicate detachment of the dead cells. **(B)** Lung morphology was evaluated using a lung injury scoring system. **(C)** Lung tissue oxidative damage was detected with in situ OxyIHC oxidative stress detecting assay. **(D)** Lung tissue cell death was detected with *in situ* TUNEL assay. **(E)** The number of TUNEL^+^ cells. The concentration of **(F)** TNF-α, **(G)** IL-1b, and **(H)** HMGB-1 in the DCD lung graft tissue was assessed by ELISA. Lung grafts from rats that received PBS vehicle (Ve) or HMGB-1 (H) were extracted after 40 min of cardiac arrest and stored in the 4 °C UW solution for 16 h (CI16). **(I)** Histology (H&E staining) of the lung graft. **(J)** Lung injury score. Scale bar: 50 μm. Data are presented as scatter plot and expressed as median with interquartile range. n = 6. *p < 0.05, **p < 0.01, ***p < 0.001, ****p < 0.0001.

When compared with non-cold preserved lung grafts from living donors (LD CI0h), cold preserved LD lung grafts (LD CI16h) stained with H&E had areas with a lighter eosinophilic coloured cytoplasm and exfoliated nucleus and an increased lung injury score (p < 0.001), indicating cold ischaemia injury. Warm ischaemia before graft extraction (DCD CI0h) significantly increased the lung injury score (p < 0.001), and the following cold preservation (DCD CI16h) exacerbated the lung graft injury (p < 0.05), as evidenced by light pink coloured and faded cytoplasm and nuclei, indicating heave leakage of protein structures and coagulative necrosis (Figure 1A-B). Immunohistochemical staining with OxyIHC (Figure 1C) showed that cold preservation slightly increased oxidative stress in LD lung grafts (LD CI16h), whereas induced severe oxidative damage in DCD lung grafts (DCD CI16h). Lung alveolar apoptotic cell death was also increased after cold preservation both in the LD (LD CI16h; p < 0.0001) and DCD (DCD CI16h; p < 0.001) lung grafts, while DCD lung grafts had more apoptotic cells (Figure 1D-E). Additionally, cold preservation caused a significant increase in DCD lung tissue cytokine production, including TNF-α (p < 0.05), IL-1β (p < 0.05), and HMGB-1 (p < 0.01) (Figure 1F-H). Histological analysis exhibited an increase in both lung parenchymal damage (Figure 1I) and injury score (Figure 1J; p < 0.01) when cold-preserved DCD lung grafts were challenged with HMGB-1.

### Dex supplementation suppresses necroptosis and inflammation in DCD lung grafts

**Figure 2:**
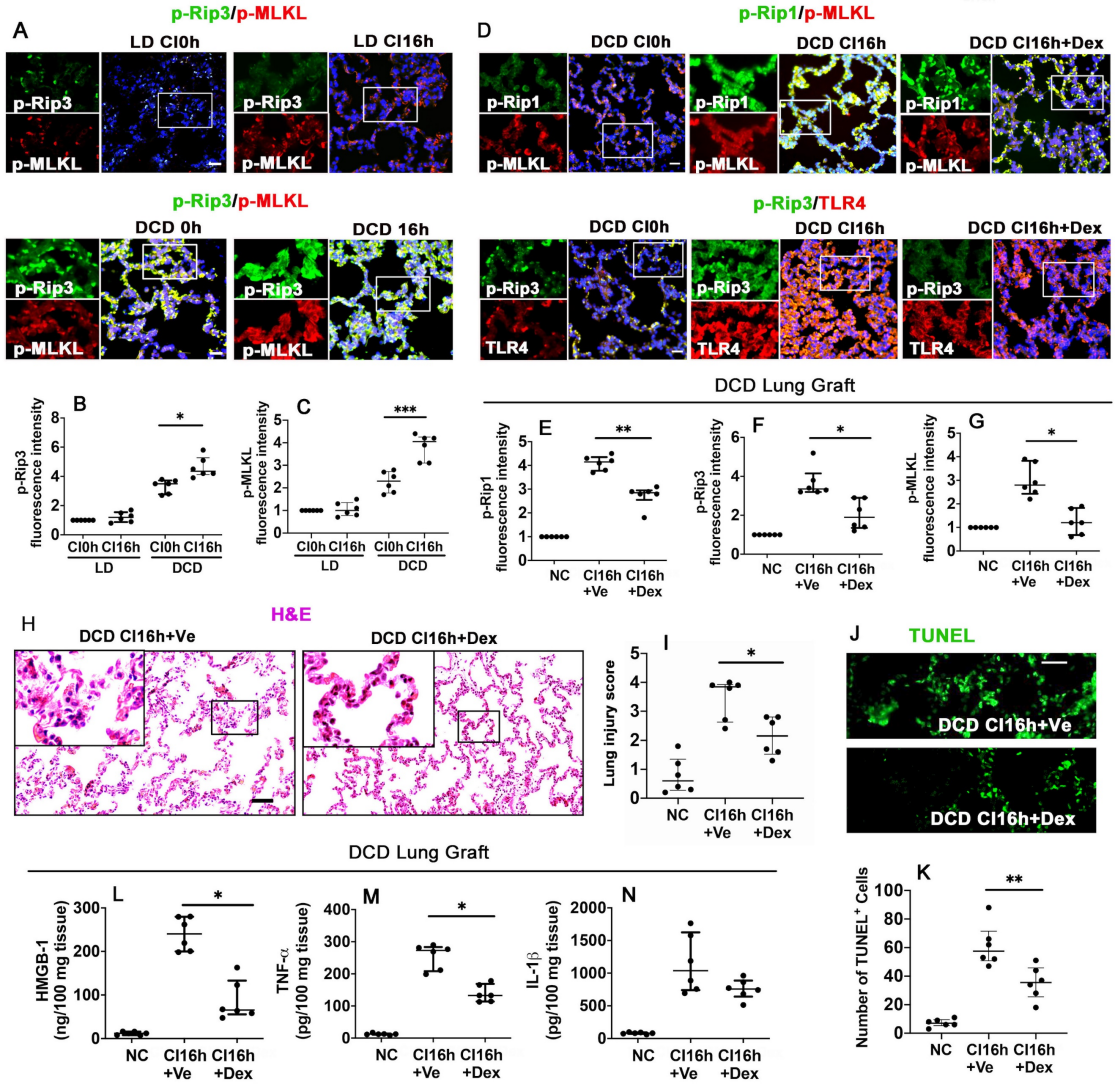
** Dex supplementation suppresses necroptosis and inflammation in DCD lung grafts.** Lung grafts were extracted from living donation (LD) and donation of cardiocirculatory death (DCD) donor rats and subsequently stored in a 4 °C preservation solution for 0 or 16 h (CI0h or CI16h). Labelling of (**A**) p-Rip3 (green) and p-MLKL (red) and fluorescent intensity of (**B**) p-Rip3 and **(C)** p-MLKL in lung tissues. Lung grafts were extracted after 40 min of DCD donors and stored in a 4 °C cold preservation solution supplemented with Dex (0.1 nM) or PBS (Ve) for 16 h (CI16h). Dual labelling of **(D)** p-Rip1 (green) and p-MLKL (red), p-Rip3 (green) and TLR-4 (red) in lung tissues. Fluorescent intensity of **(E)** p-Rip1, **(F)** p-Rip3, and **(G)** p-MLKL in lung tissues. **(H)** Histology (H&E staining) of lung grafts from DCD rats. **(I)** Lung morphology was evaluated using a lung injury scoring system. **(J)** Lung tissue cell death was detected with an *in situ* TUNEL assay. **(K)** The number of TUNEL^+^ cells. The concentration of **(L)** HMGB-1, **(M)** TNF-α, and **(N)** IL-1β in lung tissues were assessed by ELISA. Nuclei were counterstained with 4',6-diamidino-2-phenylindole (DAPI) (blue). Scale bar: 50 μm. Data are presented as scatter plot and expressed as median with interquartile range. n = 6. *p < 0.05, **p < 0.01, ***p < 0.001. The smaller box view in the merged image with three immunofluorescent channels (right) was enlarged and presented with two-channel images individually on the left of each panel.

To identify the impact of warm ischaemia on DCD lung graft injury, lung grafts from LD and DCD donors were assessed with necroptosis markers, p-Rip3 and p-MLKL (Figure 2A). Results showed that warm ischaemia increased p-Rip3 and p-MLKL expression, which continued to increase after cold storage in DCD lung grafts (p < 0.05, p < 0.001, respectively). These changes in p-Rip3 and p-MLKL expression were not found in the LD lung grafts without prior warm ischaemic insult (LD CI16h), indicating that warm ischaemia may be responsible for initiating necroptosis (Figure 2B-C). Additionally, DCD lung grafts preserved in the preservation solution supplemented with Dex (DCD CI16h+Dex) significantly reduced the expression of necroptosis biomarkers p-Rip1 (Figure 2D-E; p < 0.01), p-Rip3 (Figure 2D, F; p < 0.05), and p-MLKL (Figure 2D, G; p < 0.05), in addition to a reduction in TLR-4 activation. A remarkable improvement in lung morphology (Figure 2H-I; p < 0.05) and a reduction in apoptotic cells TUNEL^+^ cells (Figure 2J-K; p < 0.01) were also observed when the DCD lung graft was preserved in Dex-supplemented preservation solution. As a result, the concentration of HMGB-1 (Figure 2L; p < 0.05) and TNF-α (Figure 2M; p < 0.05) was significantly decreased. Ferroptosis was also evaluated after cold preservation (Figure S1); however, the ferroptosis marker, GPX4, exhibited no significant changes in the LD or DCD lung grafts or human lung epithelial A549 cells after cold preservation (Figure S1A-E).

### Dex activates the Nrf-2 pathway and suppresses oxidative stress in DCD lung grafts

**Figure 3:**
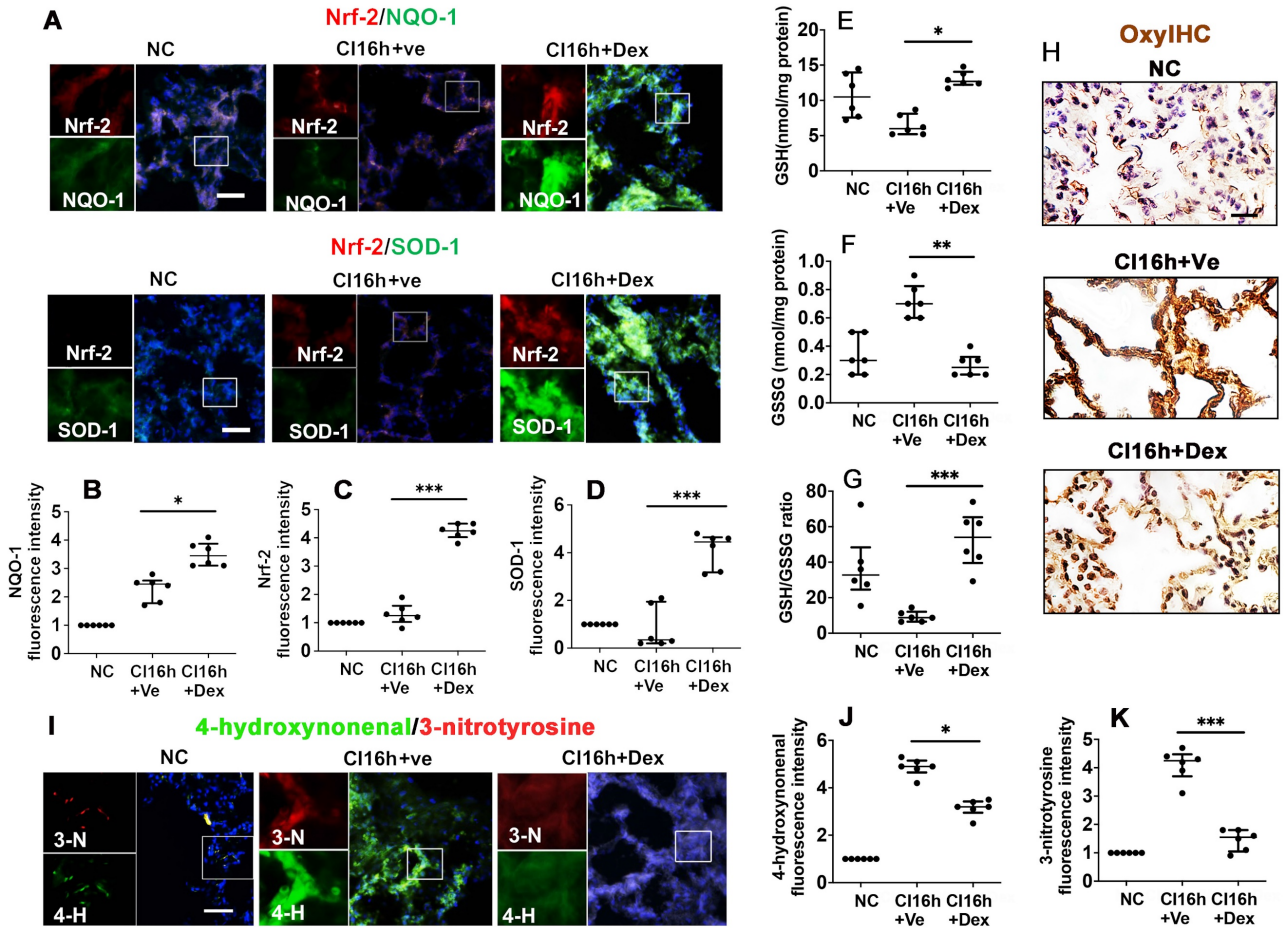
** Dex activates the Nrf-2 pathway and suppresses oxidative stress in DCD lung grafts.** Lung grafts were extracted after 40 min of cardiocirculatory death (DCD) and stored in a 4 °C cold preservation solution supplemented with Dex (0.1 nM) or PBS (Ve) for 16 h (CI16h). Normal lung grafts served as the naive control (NC). Dual labelling of **(A)** NQO-1 (green) and Nrf-2 (red), SOD-1 (green) and Nrf-2 (red) in lung tissue. Fluorescence intensity of **(B)** NQO-1, **(C)** Nrf-2, and **(D)** SOD-1 in lung tissue. **(E)** Lung tissue GSH level, **(F)** lung tissue GSSG level. **(G)** Lung tissue GSH to GSSG ratio. **(H)** Oxidative damage was evaluated by OxyIHC oxidative stress detecting assay. **(I)** The expression of 4-hydroxynonenal (green) and 3-nitrotyrosine (red) was assessed with immunofluorescent labelling. Fluorescent intensity of **(J)** 4-hydroxynonenal and **(K)** 3-nitrotyrosine. Nuclei were counterstained with 4',6-diamidino-2-phenylindole (DAPI) (blue). Scale bar: 50 μm. Data are expressed as a scatter plot, median with interquartile range. n = 6. *p < 0.05, **p < 0.01, and ***p < 0.001. The smaller box view in the merged image with three immunofluorescent channels (right) was enlarged and presented with two-channel images individually on the left of each panel.

Nrf-2 and its downstream effectors, NQO-1 and SOD-1, are critical antioxidant enzymes involved in dampening the effects of the pro-inflammatory response during IR injury. Therefore, we assessed the impact of Dex on the activation of these protective mediators. DCD lung grafts stored in Dex-supplemented preservation solution (DCD CI16h+Dex) experienced a significant increase in the expression of NQO-1 (Figure 3A-B; p < 0.05), Nrf-2 (Figure 3A, C; p < 0.001), and SOD-1 (Figure 3A, D; p < 0.001). Reduced glutathione (GSH) is a critical scavenger of reactive oxygen species, whilst its ratio with oxidised glutathione (GSSG) is a commonly used marker of oxidative stress. Lung grafts stored in Dex-supplemented cold preservation solution experienced a significant increase in the expression of GSH (Figure 3E; p < 0.05) and a significant decrease in GSSG (Figure 3F; p < 0.01), and the ratio of GSH/GSSG was increased (Figure 3G; p < 0.001). A reduced signal for OxyIHC oxidative stress staining was also observed in grafts preserved in the Dex-supplemented solution (Figure 3H). 3-nitrotyrosine is a marker of nitrogen-free radical species, whilst 4-hydroxynonenal is an unsaturated hydroxyalkenal produced by lipid peroxidation in cells. The expression of both 4-hydroxynonenal (Figure 3I-J; p < 0.05) and 3-nitrotyrosine (Figure 3I, K; p < 0.001) was significantly reduced in lung grafts preserved in Dex-supplemented cold preservation solution.

### Nrf-2 suppression and Atipamezole both attenuate Dex-mediated DCD lung graft protection

**Figure 4:**
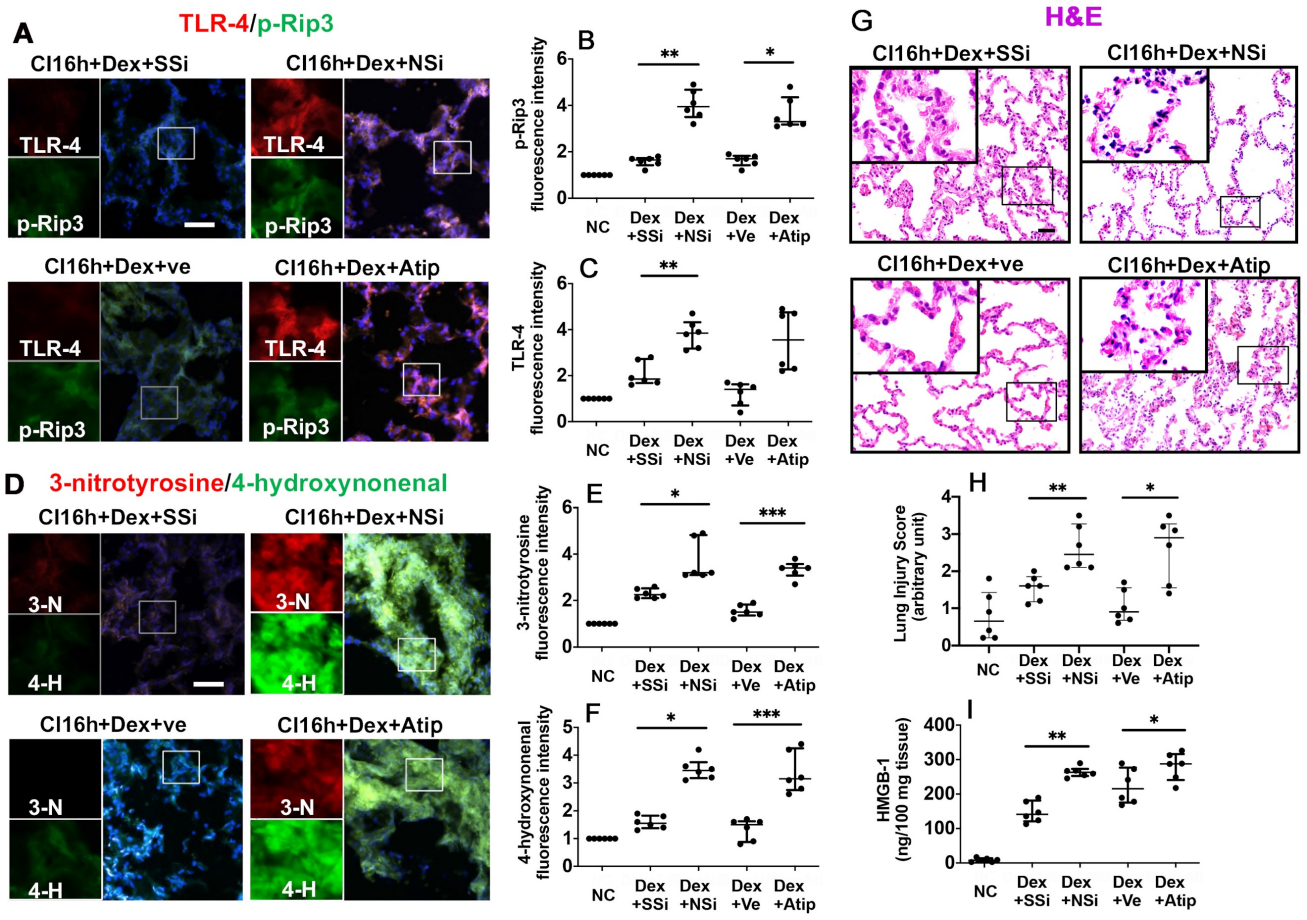
** Nrf-2 suppression and α-adrenoceptor antagonism with atipamezole attenuate Dex-mediated rat lung graft protection.** Lung grafts were extracted after 40 min of cardiocirculatory death and stored in a 4 °C UW cold storage solution saturated with Dex (0.1nM) or PBS (Ve) for 16 h (CI16h). Nrf-2 siRNA (NSi) or atipamezole (Atip) was given to the donor before graft extraction in comparison with scrambled siRNA (SSi) or PBS (Ve), respectively. Dual TLR-4 (red) and p-Rip-3 (green) were labelled in lung tissue after **(A)** siRNA or Atip treatment. Fluorescence intensity of **(B)** p-Rip3 and **(C)** TLR-4 in lung tissue. **(D)** The expression of 4-hydroxynonenal (green) and 3-nitrotyrosine (red) was assessed with immunofluorescent labelling. The expression of **(E)** 3-nitrotyrosine and **(F)** 4-hydroxynonenal was assessed. **(G)** Histology (H&E staining) of the lung graft and **(H)** injury score. **(I)** HMGB-1 in lung graft tissue was assessed by ELISA. Nuclei were counterstained with 4',6-diamidino-2-phenylindole (DAPI) (blue). Scale bar: 50 μm. Data are expressed as scatter plot, median with interquartile range. n = 6. *p < 0.05, **p < 0.01 and ***p < 0.001. The smaller box view in the merged image with three immunofluorescent channels (right) was enlarged and presented with two-channel images individually on the left of each panel.

The administration of Nrf-2 siRNA to donor rats before lung extraction (DCD CI16h+Dex+NSi) reversed the protective effects of Dex by enhancing the expression of p-Rip3 and TLR-4 (Figure 4A-C; p < 0.01, respectively). Additionally, administration of atipamezole, an α_2_-adrenergic receptor antagonist (DCD CI16h+Dex+Atip), was associated with enhanced expression of p-Rip3 (Figure 4A-B, p < 0.05). Nrf-2 siRNA and atipamezole treatment both resulted in enhanced oxidative stress, as evidenced by a significant increase in 3-nitrotyrosine (Figure 4D-E; p < 0.05 and p < 0.001, respectively) and 4-hydroxynonenal expression (Figure 4D, F; p < 0.05 and p < 0.001, respectively). Furthermore, DCD lung grafts treated with either Nrf-2 siRNA or atipamezole demonstrated an increased lung injury score (Figure 4G-H; p < 0.01 and p < 0.05, respectively) and HMGB-1 release (Figure 4I; p < 0.01 and p < 0.05, respectively).

### Dex supplementation protects human lung tissue during cold storage

**Figure 5:**
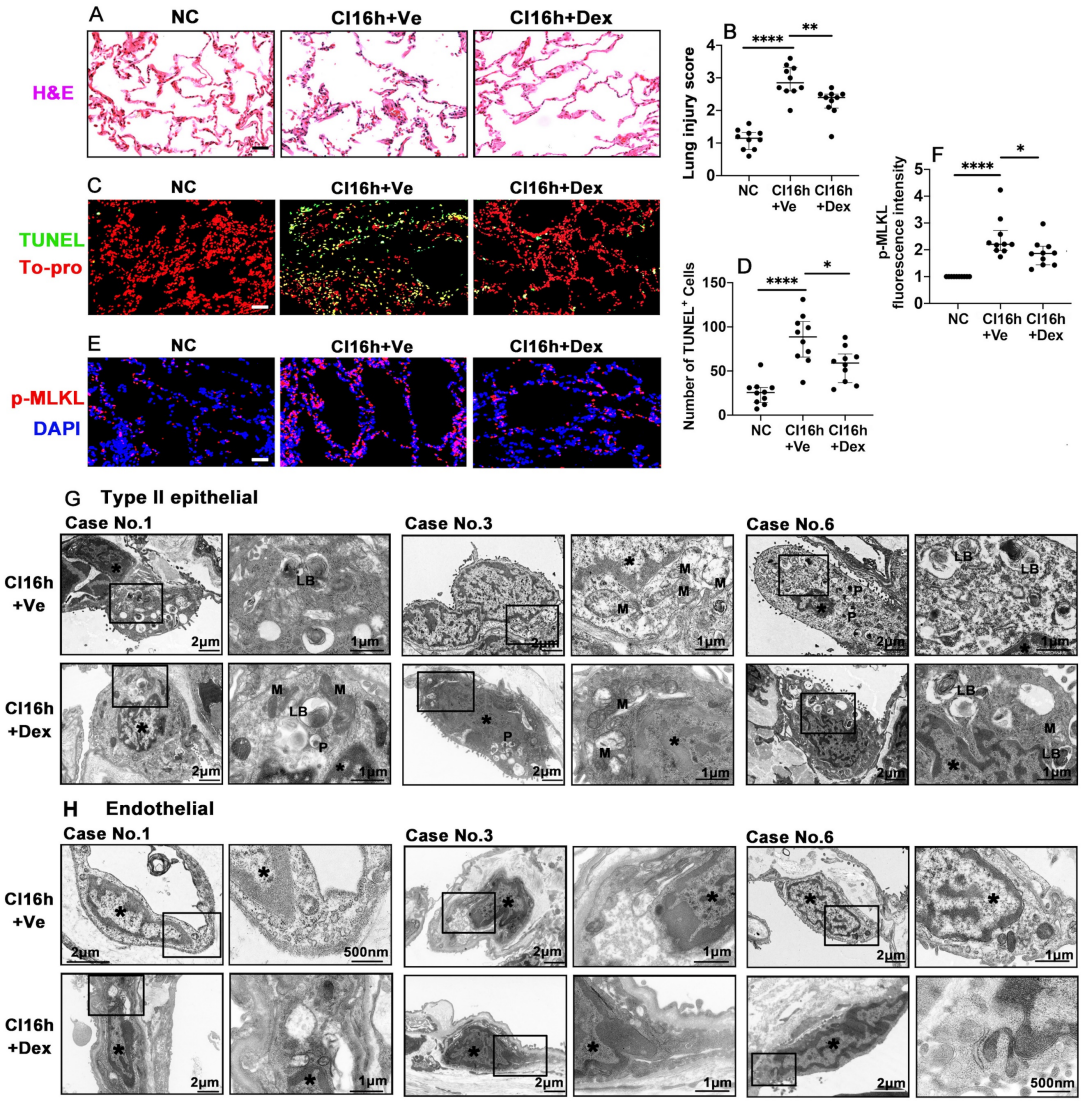
** Dex supplementation protects human lung tissue during cold storage.** Lung tissues from patients were cut into 3 pieces, 2 pieces were stored in a 4 °C UW solution saturated with Dex (0.1 nM) or PBS (Ve) for 16 h (CI16h) separately; another piece served as the naive control (NC). **(A)** Histology (H&E staining) of the lung tissue. **(B)** Lung morphology was evaluated using a lung injury scoring system. **(C)** Lung tissue cell death was detected by in situ TUNEL assay. **(D)** The number of TUNEL^+^ cells. Labelling of **(E)** p-MLKL (red) and fluorescent intensity of **(F)** p-MLKL in lung tissue. Nuclei were counterstained with 4',6-diamidino-2-phenylindole (DAPI) (blue) or To-pro (red). Scale bar: 50 μm. Data are presented as scatter plots and expressed median with interquartile range. n = 10. *p < 0.05, **p < 0.01 and ****p < 0.0001. Transmission electron microscopy was performed on lung samples from patients No. 1, 3, and 6; **(G)** type II epithelial cells and **(H)** endothelial cells were observed. Asterisk: chromatin condensation; P: phagosome; LB: lamellar body; M: mitochondrial.

We also investigated whether the therapeutic value of Dex-saturated cold preservation solution enhanced protection to lung grafts from rats is also evident in human lung tissues. Cold storage with Dex-saturated solution significantly improved human lung morphology (Figure 5A) and reduced the lung injury score (Figure 5B; p < 0.01). Dex supplementation also reduced the number of TUNEL^+^ cells (Figure 5C-D; p < 0.05) and the expression of necroptosis biomarker p-MLKL (Figure 5E-F; p < 0.05). Additionally, cold storage caused the marginalisation of nuclear chromatin and moderate dilatation of the endoplasmic reticulum (ER) and perinuclear cisternae in both type II epithelial cells (Figure 5G) and endothelial cells (Figure 5H). Mitophagosome and phagosome formation were also increased. The apical cell surface lost massive microvilli, while visible lamellar bodies (LB) were reduced in type II epithelial cells. The epithelial and endothelial injury was reduced when the Dex-saturated UW solution was applied in cold storage, although due to low N number and semi-quantitive nature, it did not reach statistical differences (Figure 5G-H and Table S2). In addition, there was no change in the expression of the ferroptosis marker, GPX4, in human lung tissues after being preserved in the Dex-saturated solution (Figure S2A-D).

### Hypothermic hypoxia-Reoxygenation (HR) challenge induces necroptosis and plasma membrane rupture

**Figure 6:**
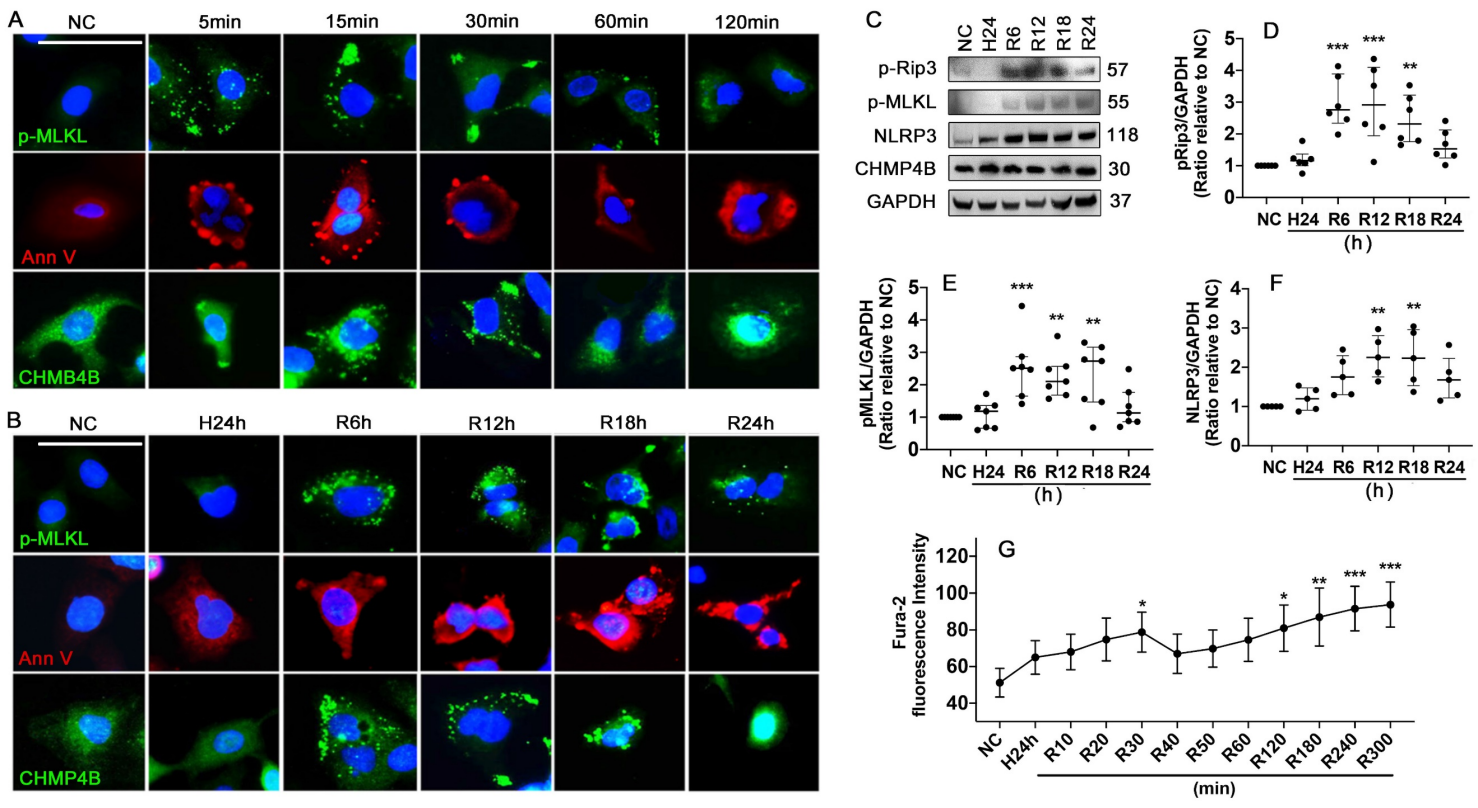
** Hypothermic hypoxia-reoxygenation (HR) challenge induces necroptosis and plasma membrane rupture.** Human lung epithelial A549 cells were preserved in a 0-4 °C UW solution for 24 h (H24h) to simulate hypothermia/hypoxia condition, then re-cultured in 37 °C fresh media to simulate reoxygenation. Labelling of p-MLKL (green), Annexin V (red), and CHMP4B (green) in A549 cells treated with **(A)** TLQ (TNF- α, 150 ng/mL; Q-VD-Oph, 40 µM; and LCL161, 10 µM) or **(B)** HR challenge. Nuclei were counterstained with 4',6-diamidino-2-phenylindole (DAPI) (blue). Scale bar: 10 μm. **(C)** Western blot showing analysis **(D)** p-Rip3, **(E)** p-MLKL, and **(F)** NLRP3, GAPDH as a loading control. n = 5-7. A549 cells were preserved in 0-4 °C UW solution for 24 h, then probed with 2 μM calcium indicator Fura-2 for 30 min and cultured in 37 °C media. The fluorescence intensity **(G)** of Fura-2 of 90 cells was calculated in each group. n = 5. Data are presented as scatter plot and median with interquartile range, or mean ± SD. *p < 0.05, **p < 0.01, ***p < 0.001 and ****p < 0.0001.

Necroptosis and cell plasma membrane damage induced by HR challenge was assessed using human lung epithelial A549 cells. The results showed that standard necroptosis inducers of TNF-α, LCL161 and Q-VD-Oph in combination (TLQ) increased p-MLKL expression in the cells and accumulated at the plasma membrane at 5, 15, 30, 60, and 120 min (Figure 6A). HR also accumulated p-MLKL in the plasma membrane at 6, 12, and 18 h after reoxygenation (Figure 6B). Meanwhile, TLQ treatment induced visible Annexin V^+^ vesicles that were released from the cell membrane within 60 min, and significant membrane pores were detected at 120 min (Figure 6A). While the HR challenge was associated with a much more seriously damaged plasma membrane (Figure 6B). CHMP4B is a subunit of ESCRT-III that can transfer to the cellular membrane wound and participate in the formation of membrane vesicles. TLQ treatment and HR promoted CHMP4B clustering to the cell plasma membrane, participating in membrane repair (Figure 6A-B). In addition, HR challenge significantly enhanced necroptosis by up-regulating the expression of p-Rip3 (Figure 6C-D) and p-MLKL (Figure 6C, E), as well as promoting NLRP3 expression (Figure 6C, F) in a time-dependent manner. Intracellular Ca^2+^ concentration was also increased during the HR challenge, and double peaks at 30 (p < 0.05) and 300 min (p < 0.001) were found after reoxygenation (Figure 6G), suggesting that the cells may capture Ca^2+^ from the extracellular and release it from its intracellular storage.

### Dex supplementation reduces oxidative stress and PANoptosis after hypothermic hypoxia-reoxygenation (HR)

**Figure 7:**
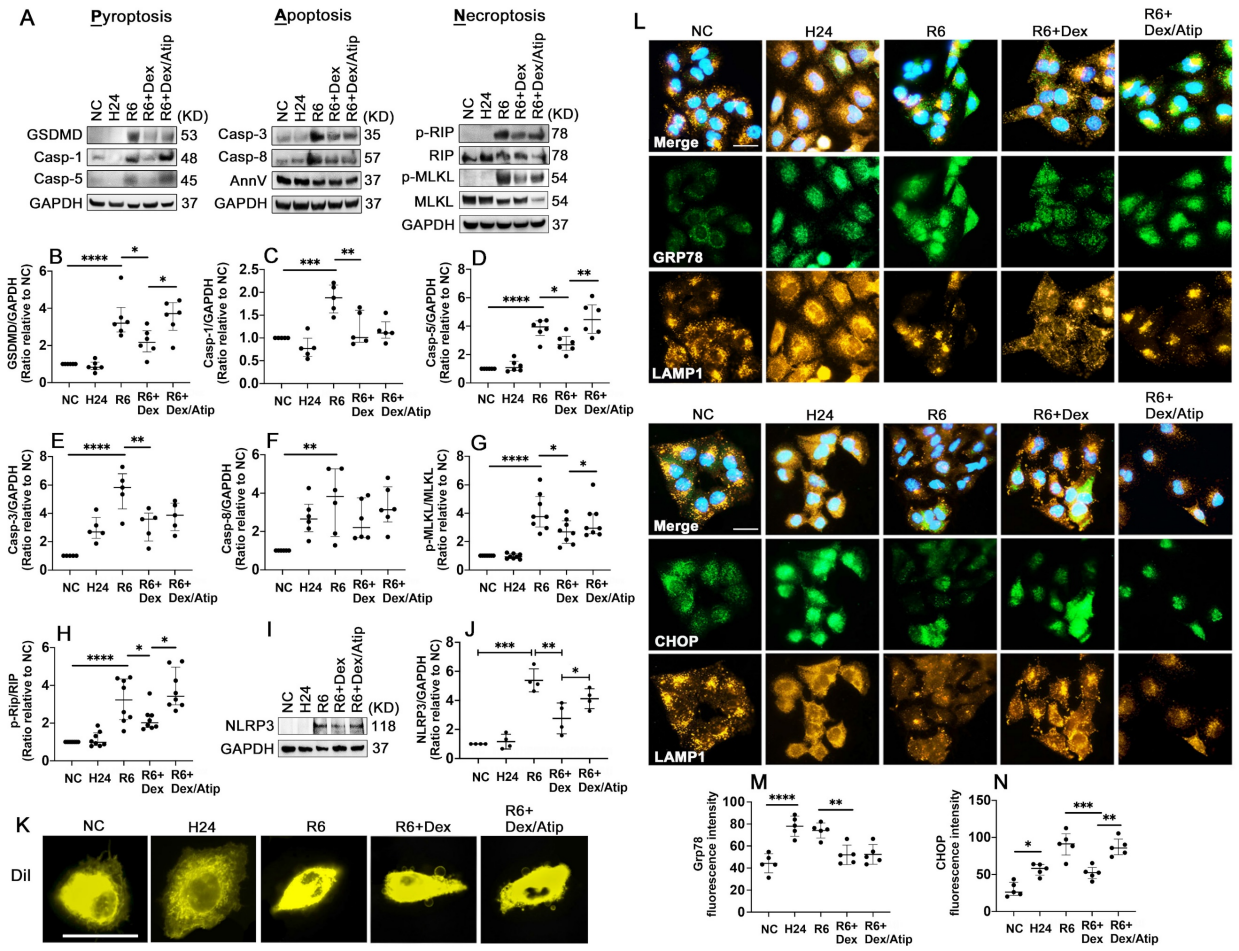
** Dex supplementation reduces oxidative stress and PANoptosis after hypothermic hypoxia-reoxygenation (HR).** A549 cells were stored in a 4 °C UW solution saturated with or without Dex (0.1 nM) or /and atipamezole (Atip) for 24 h and were then cultured in 37 °C media for another 6 h. **(A)** Western Blot analysis of **(B)** GSDMD, **(C)** Caspase-1 (Casp-1), **(D)** Caspase-5 (Casp-5), **(E)** Caspase-3 (Casp-3), **(F)** Caspase-8 (Casp-8), **(G)** p-MLKL, **(H)** p-Rip, **(I-J)** the expression of NLRP3 in A549 cells experienced HR.** (K)** Membrane lipid was stained by DilC18 dye. **(L)** Dual labelling of LAMP1 (yellow) and Grp78 (green), LAMP1 (yellow) and CHOP (green) in A549 cells. Nuclei were counterstained with 4',6-diamidino-2-phenylindole (DAPI) (blue). Fluorescence intensity of **(M)** Grp78 and **(N)** CHOP in A549 cells. Data are presented as scatter plot and expressed as median with interquartile range. Scale bar: 10 μm. n = 4-8. *p < 0.05, **p < 0.01, ***p < 0.001, **** p < 0.0001.

HR significantly activated Gasdermin D (GSDMD) (Figure 7A-B; p < 0.0001), casp-1 (Figure 7A, C; p < 0.001), and casp-5 (Figure 7A, D; p < 0.0001), which may induce pyroptosis. HR also induced apoptosis by activating apoptotic caspases, casp-3 (Figure 7A, E; p < 0.0001) and casp-8 (Figure 7A, F; p < 0.01). Robust phosphorylation of MLKL (Figure 7A, G; p < 0.0001) and Rip (Figure 7A, H; p < 0.0001) were also detected after HR stimulation. UW solution saturated with Dex significantly decreased these cell deaths through reducing the activation of GSDMD (p < 0.05), casp-1 (p < 0.01), casp-5 (p < 0.05), casp-3 (p < 0.01), p-MLKL (p < 0.05), and p-Rip (p < 0.05); whilst this reduction was partially reversed by co-saturation with atipamezole. Additionally, the cells treated with the Dex-saturated UW solution significantly reduced NLRP3 expression (Figure 7I, J; p < 0.01) and improved outer membrane vesicle formation (Figure 7K). Meanwhile, hypothermia/hypoxia resulted in enhanced ER stress by increasing the expression of Grp78 (Figure 7L-M; p < 0.0001) and CHOP (Figure 7L, N; p < 0.05). Cells preserved in Dex-saturated UW solution exhibited a reduction in Grp78 (Figure 7L-M; p < 0.01) and CHOP (Figure 7L, N; p < 0.001) expression. Lysosomal-regulated ER turnover prevents excessive ER expansion and degrades unfunctional and unfolded proteins during ER stress. Thus, lysosomal-associated membrane protein 1 (LAMP-1) was double stained with Grp78 or CHOP to evaluate lysosomal function (Figure 7L). Small round lysosomes located around the edge of the nucleus were detected, while a small number of lysosomes were scattered in the cytoplasm in the control group. After hypoxia, lysosomes were distributed throughout entire cells with a tiny and dense structure, indicating lysosomal rupture. Cells preserved in Dex-saturated solution maintained the normal structure of lysosomes during hypoxia. Additionally, after reoxygenation, features of cell death were characterised by cell swelling and paucity of lysosomes, whilst Dex treatment maintained the volume and morphology of lysosomes.

## Discussion

**Figure 8:**
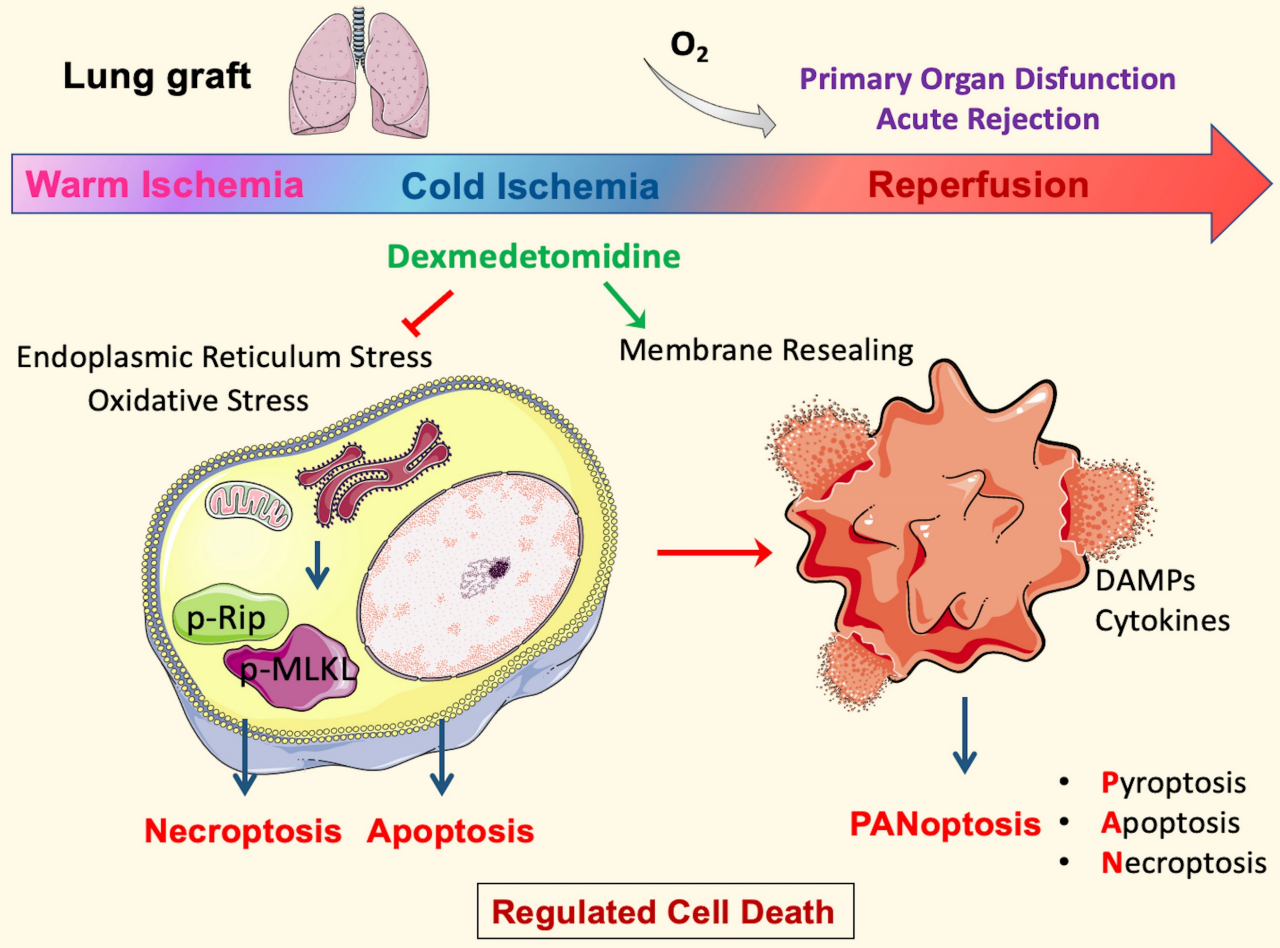
** Molecular mechanisms of Dexmedetomidine-mediated protection to lung grafts.** In the DCD setting, the inevitable warm ischemia during cardiocirculatory arrest and organ extraction leads to endoplasmic reticulum stress and oxidative stress that initiates necroptosis and apoptosis. The following cold storage means a lack of ATP production by oxidative phosphorylation. However, ATP demanding process continues because cold tolerance is finite; metabolism may slow but does not entirely stop at low temperatures. These will cause energy depletion, and hence ions to pump failure exacerbate cell death. Furthermore, the damage to cells upon reperfusion releases damage-associated molecular patterns (DAMPs) and pro-inflammatory cytokines, which initiate PANoptosis. Pore-forming proteins, such as GSDMD and MLKL in PANoptotic cells, lead to plasma membrane rupture and allow cytoplasmic outflow, which activates the innate immune response and enhances inflammatory damage to the tissue, then contributes to organ dysfunction and rejection. Dexmedetomidine (Dex) treatment in the ex vivo cold-storing stage inhibited oxidative stress and the following necroptosis. In addition, Dex can stabilise lysosomes during cold storage and promote membrane repair after reperfusion, maintain cell membrane integrity, and suppresses PANoptosis core proteins. These protective effects preserve lung epithelial cells and DCD lung graft function after engraftment *per se*.

This study, for the first time, demonstrated that cold preservation solution supplemented with Dex conferred significant protection against lung graft ischaemic damage by attenuating regulated cell death and the subsequent inflammatory response. Dex exerted a remarkable therapeutic effect by attenuating the activation of necroptotic execution proteins and inflammatory cytokine production, thus significantly improving the morphology of cold-preserved lung grafts. Furthermore, the antioxidative effects of Nrf-2 activation were also at least partially responsible for the protective effects conferred by Dex. Additionally, necroptosis and cell membrane rupture occurred in lung epithelial cell cultures induced by hypothermic hypoxia reoxygenation challenge, whilst Dex promoted cell membrane repairing function to reseal the damaged cell membrane. The multiple protective effects described above, in addition to the reduction in endoplasmic reticulum stress, lysosome damage, and inflammation, promoted an improvement in lung graft viability (summarised in Figure 8).

Necroptosis plays a critical role in ischaemia injury in an experimental rat model of DCD lung graft preservation. We found that necroptosis was not detected in lung grafts from living donor rats following cold preservation; this may be due to the fact that the process of regulated cell death is highly energy- and enzyme-dependent 28. Nevertheless, lung grafts from DCD donors were excised after 40 min of cardiac arrest; this hypoxia and hypotension situation led to systemic hemodynamic disturbances and metabolic abnormalities, resulting in lung dysfunction and necroptosis initiation, as cardiac arrest represents the most severe form of shock 29. Subsequent cooling and cold preservation may exacerbate necroptotic cell death because cold is a damaging insult, and metabolism may slow down but does not stop at low temperatures 30. In total, our data indicated that the duration of warm ischaemia might play a more important role in influencing the clinical outcomes of lung transplant recipients. Cold preservation solution supplemented with Dex suppressed necroptosis and its associated deleterious sequelae during cold storage and after that *per se*.

Our study further demonstrated that the suppression of necroptosis by Dex was related to the activation of the transcription factor Nrf-2. Nrf-2 is an organoprotective mediator that attenuates oxidative stress, as demonstrated by the downregulated expression of numerous oxidative stress markers, including 3-nitrotyrosine/4-hydroxynonenal 31. Nrf-2 is a basic leucine zipper transcription factor that binds and activates the transcription of antioxidant response element (ARE) 32. Under oxidative stress, Nrf-2 translocates into the nucleus and activates its target genes-related proteins, including heme oxygenase-1 (HO-1), glutathione S-transferases (GSTs) and NAD(P)H quinone oxidoreductase. The activations result in reactive oxygen species (ROS) scavenging and decrease oxidative stress-induced tissue damage 33. In addition, a previous experimental study revealed that up-regulation of the Nrf-2-ARE pathway results in inhibitory regulation of HMGB1 secretion from macrophages/monocytes cells 34. HMGB-1 is released by cells undergoing necroptosis and activates an inflammatory response *via* interaction with TLR-4. The TLR-4 activation induces an inflammatory response, which may increase tissue damage upon reperfusion 35. Therefore, it is possible that Nrf-2 up-regulation by Dex inhibits HMGB-1 release and TLR-4 activation, subsequently dampening the inflammatory response.

Cell plasma membrane repair is a self-defence mechanism that maintains membrane integrity and prevents cell death 36. When undergoing necroptosis, p-MLKL ubiquitination results in its insertion into the plasma membrane, followed by the rapid influx of Ca^2+^ into cells, leading to phosphatidylserine exposure on the cell surface before membrane disruption 37. Subsequently, ESCRT-III machinery translocates to the damaged membrane from the cytosol, participating in membrane resealing by releasing membrane vesicles 37-39. In our *in vitro* study, phosphatidylserine exposure labelling by Annexin V was found during hypothermic preservation, whilst the formation of membrane vesicles mediated by CHMP4B (a subunit of ESCRT-III machinery) was detected after reoxygenation, suggesting that cell plasma membrane damage starts at cold storage stage and membrane resealing mainly begins at the reoxygenation stage.

Our study has several limitations. Firstly, unlike previous studies 40, 41, which are closer to the “clinical scenario”, a cancer cell line was used in our study, and hence the translational value of the current findings of the *in vitro* part is unknown. Secondly, this is just a proof-of-concept study far away from clinical settings; in particular, lung graft is normally perfused with a preservation solution before engraftment clinically. Therefore, more studies with other preservation solutions, such as Perfadex solution, and *ex vivo* perfusion towards *in vivo* settings, are urgently needed before clinical study/trial can be launched 42, 43. Thirdly, we evaluated PANoptosis in the *in vitro* hypothermic hypoxia reoxygenation model. It was suggested that the existence of PANoptosis-like cell death could be either pyroptosis, apoptosis, or necroptosis. Therefore, further studies are required to investigate the existence of the PANoptosome that simultaneously regulates these three regulated cell deaths. Lastly and importantly, a large amount of the current work was based on lung tissues from rats; due to technical challenges, the lung transplant can not be performed in this species, and therefore, the key elements in the transplant setting, for example, the alloimmune response including immune cell subset changes, immune cell infiltration and graft functions are largely unknown. All these need to be determined in large animals, e.g. pigs, in future studies superposed with the novel strategy reported here. In addition, the overall production of IL-1β and TNF-α was reduced by Dex treatment. The production of pro-inflammatory cytokines might be mostly triggered by immune cells present in the DCD lung grafts during warm ischaemia, which might be aggregated by progressive necrosis development during the cold ischaemia stage 44. Therefore, our data provided further evidence that Dex actually restrained immune cell-derived cytokine production, either through inhibiting regulated cell necrosis or direct anti-inflammatory effects on immune cells. However, a more profound analysis of the inflammatory response during and after cold storage in Dex-treated lungs, especially the treatment effects on distinct immune cell subsets (such as monocytes/macrophages), should be considered in future studies to provide more evidence to support the conclusion.

Nevertheless, our study likely has significant clinical implications. Given the increasing reliance on marginal donors to meet the growing demand for transplantation, it is vital to refine graft preservation strategies, as the marginal organs are more susceptible to the impact of cold preservation 45. The addition of Dex to lung graft preservation solutions reduces IR injury in DCD lung grafts, resulting in improved graft function and reduced inflammation. Indeed, in recent years, *ex vivo* perfusion systems have been demonstrated to be a suitable interface to apply pharmacological drugs during the preservation 46; practical application of Dex in these systems might potentially benefit transplant patients by further improving graft quality during the preservation period. Although this preserving strategy may enhance the potential lung donor pool and the number of available lung grafts, caution to be taken is that this is a pure proof of concept study which needs to be validated in a more closely clinical setting.

## Conclusion

In summary, our data demonstrate that adding Dex to the UW preservation solution likely reduces IR injury in DCD lung grafts, resulting in improved graft function and reduced inflammation. This strategy is straightforward, can be easily applied within the clinical setting, and very likely improves lung graft quality and function in the early postoperative period, thus improving long-term patient outcomes and enhancing the donor pool of lung grafts, although it is subjected to further determination in lung transplant settings in large animals, e.g. pigs, before clinical trials can be launched.

## Supplementary Material

Supplementary figures and tables.

## Abbreviations

Atip: Atipamezole
ATP: Adenosine triphosphate
DAMPs: damage-associated molecular patterns
Dex: Dexmedetomidine
DCD: Donation after cardiac death
ER: Endoplasmic reticulum
GSH: Glutathione
GSSG: Glutathione
HR: Hypothermia/hypoxia-reoxygenation
IRI: Ischaemia reperfusion injury
LB: Lamellar bodies
LD: Living donation
PGD: Primary graft dysfunction
ROS: Reactive oxygen species
Nrf2: The nuclear factor erythroid 2-related factor 2
UW solution: University of Wisconsin solution
