# Supplementary material for: Tackling regulated cell death yields enhanced protection in lung grafts
Source: Theranostics. 2023 Jul 31;13(13):4376–90. doi: 10.7150/thno.87375 (PMC10465232; doi:10.7150/thno.87375)
Supplement: Supplementary file 1 — Supplementary figures and tables. [file thnov13p4376s1.pdf]

Supplementary Materials for

Tackling regulated cell death yields enhanced protection in lung grafts

Qian Chen, Xiangfeng Liu, Zhiheng Liu, Shujing Zhang, Lin Chen, Shiori Eguchi, Azeem Alam, Zhen Cahilog, Qizhe Sun, Lingzhi Wu, Enqiang Chang, Zhiping Wang, Jianteng Gu, Hailin Zhao and Daqing Ma

This file includes:

Tables. S1 to S2

Figures. S1 to S2

Table S1. Clinical characteristics of patients (n = 10)

Case No.	1	2	3	4	5	6	7	8	9	10	Mean ± SD or Median (IQR)
**Age, (years)**	54	50	55	46	54	45	57	52	50	56	52 ± 4
**BMI, (kg/m^2)**	21	25	20	23	24	25	20	25	20	20	22 ± 2
**Pre-operation PO_2:FiO_2 ratio**	391	413	412	376	419	507	475	573	442	480	450 ± 60
**Warm ischemia time, (min)**	19	44	40	12	12	59	102	13	17	42	29.5 (31)

Table S2. Electron microscopy statistical analysis results (n = 3)

**Type II epithelial cells**	**Mean ± SD**		**P value**
	**CI16+Ve**	**CI16+Dex**	
**Number of phagosome and mitophagosome-like structure**	8.33 ± 3.51	6.67 ± 2.52	0.54
**Ratio of damaged mitochondrial (%)**	45.44 ± 11.50	35.37 ± 6.20	0.26
**Number of visible lamellar bodies**	34.33 ± 6.11	42.67 ± 8.62	0.24

**Endothelial cells**	**Mean ± SD**		**P value**
	**CI16+Ve**	**CI16+Dex**	
**Number of phagosome and mitophagosome-like structure**	4.81 ± 1.55	3.37 ± 0.83	0.23
**Ratio of damaged mitochondrial**	45.30 ± 9.63	31.53 ± 5.10	0.09

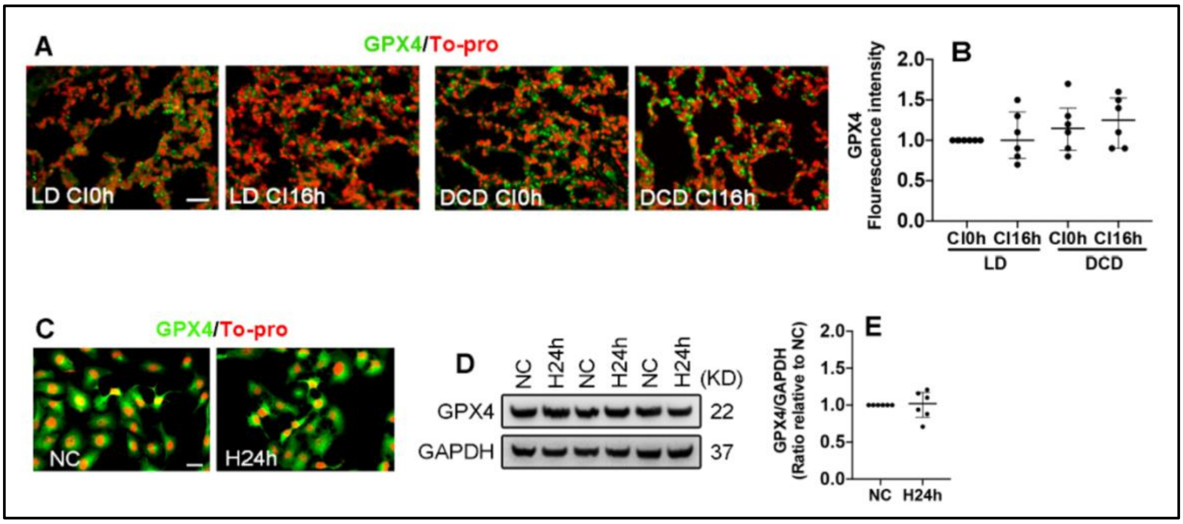

Figure S1. Cold storage did not initiate ferroptosis in lung grafts. Lewis rat lung grafts were extracted from living donors (LD) and the donation of cardiac death (DCD) donors and then stored in 4 °C UW solution for 0 or 16 hours (lung graft cold ischemia CI0h or CI16h). Labeling of (**A**) GPX4 (green) and qualified fluorescent intensity (**B**) of GPX4 in lung tissue. Human lung epithelial A549 cells were preserved in 0-4 °C UW solution for 24 hours (H24h) to simulate hypothermia/hypoxia. (**C**) Labeling GPX4 (green) and western blot analysis **(D, E)** of GPX4 in A549 cells. Nuclei were counterstained with 4',6-diamidino-2-phenylindole (DAPI) (blue) or To-pro (red). Scale bar: 50 μm (lung tissue) or 5μm (cells). Data are presented as scatter plots and expressed as median with an interquartile range (n = 6).

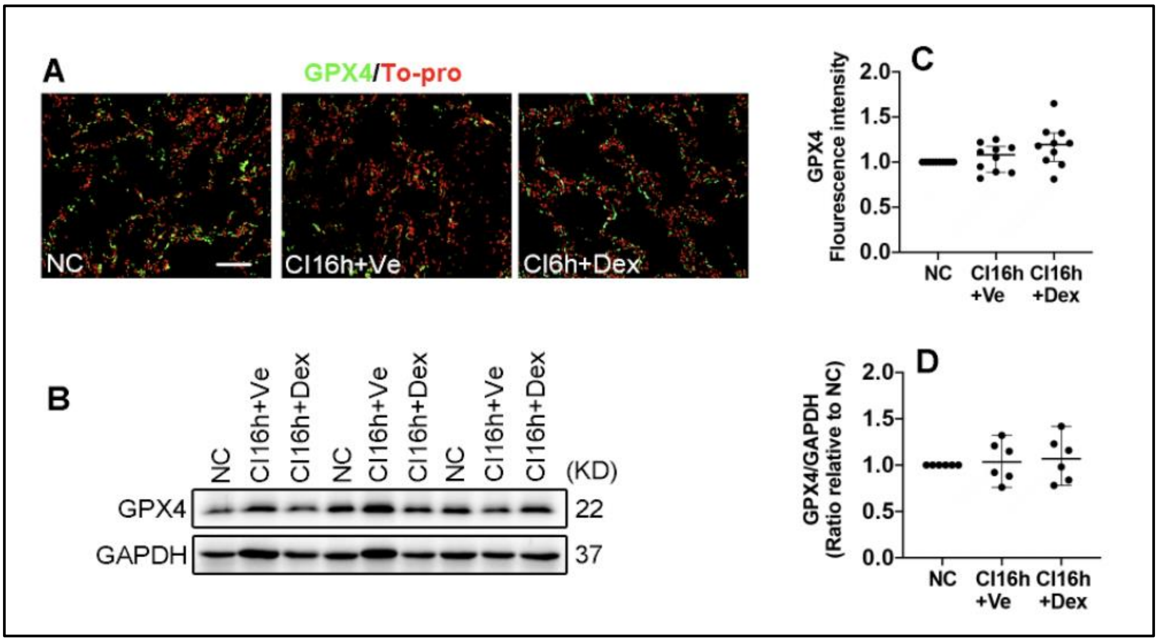

Figure S2. Dex supplementation did not affect GPX4 expression during cold storage. Lung tissues were collected from 10 female patients and stored in 4 °C UW solution saturated with Dex (0.1 nM) or PBS (Ve) for 16 hours (lung graft cold ischemia CI16h) or without challenge served as the naive control (NC). GPX4 expression was evaluated by **(A)** fluorescent staining and its intensity (**C)** and western blot analysis **(B, D)** of GPX4 in human lung tissue (n = 10). Nuclei were counterstained with To-pro (red). Scale bar: 50 μm.
